# T-wave Inversions in Precordial Leads: A Case Study of Arrhythmogenic Right Ventricular Cardiomyopathy in a Patient With Recurrent Syncope

**DOI:** 10.7759/cureus.43201

**Published:** 2023-08-09

**Authors:** Conner W Dunbar, Monica Whaley, Erin Park, Javier Escobar

**Affiliations:** 1 Department of Research, Alabama College of Osteopathic Medicine, Dothan, USA; 2 Emergency Medicine, Tallahassee Memorial Healthcare, Tallahassee, USA

**Keywords:** arrhythmogenic right ventricular cardiomyopathy (arvc/d), cardiac sudden death, vfib, recurrent syncope, unexplained syncope, t-wave inversion, abnormal ekg, emergency med, cardiology research, arvc

## Abstract

Arrhythmogenic right ventricular cardiomyopathy (ARVC) is a rare sudden cardiac death (SCD) syndrome characterized by ventricular arrhythmias of right ventricular (RV) origin. This case follows the presentation of ARVC in an otherwise healthy 26-year-old male. The patient was observed for one week after being admitted from the emergency department secondary to pre-syncope with pathognomonic findings on his electrocardiogram (EKG), echocardiogram, and cardiac imaging. The patient was started on beta-blockers, which ultimately he could not tolerate due to bradycardia, and the recommendation of an automatic implantable cardioverter-defibrillator (AICD) was refused. He was discharged without any complications or ventricular arrhythmias on telemetry while hospitalized.

## Introduction

Arrhythmogenic right ventricular cardiomyopathy (ARVC) is responsible for roughly 11% of sudden cardiac deaths (SCD) in young adults and athletes [1,2]. The disease is characterized by dilation of the right ventricle (RV) or fibrofatty replacement of the RV myocardium. Family history and genetic testing have been done to pinpoint the mutations that may cause ARVC. Numerous genes identified that could be responsible for encoding the desmosome components have been found to be commonly associated with ARVC, including DSC2, DSG2, DSP, JUP, PKP2, and TMEM43 [2]. RV dysfunction has been identified in the pathophysiology of numerous cardiovascular diseases, including ARVC [3].

In this case report, the patient is a 26-year-old male diagnosed with ARVC with nonspecific symptoms and no known family history of cardiac disease. The patient reported palpitations and non-exertional presyncopal/syncopal events. The physician initiated symptomatic treatment and recommended an automated implantable cardioverter-defibrillator, which was later declined. This case study will follow the patient’s case from presentation to the diagnostic findings of ARVC.

## Case presentation

A 26-year-old male with a past medical history of multiple unexplained, non-exertional presyncopal and syncopal events presented to the emergency department via Emergency Medical Services (EMS) with the complaint of palpitations and non-exertional presyncope while driving home from work. The patient presented to the same emergency department in the past with similar symptoms and was followed by cardiology without a definitive diagnosis. Upon arrival at the emergency department, the patient was awake and oriented with no active symptoms. He denied any history of tobacco or alcohol abuse. In addition, the patient did not have any significant findings or abnormalities on the physical exam. He was normotensive, maintained a normal sinus rhythm, and was 95% or higher on room air in the field and throughout his stay in the emergency department. The emergency physician ordered numerous labs, a chest X-ray, and an electrocardiogram (EKG). Urinalysis was negative for infection and negative for any illicit drugs, including cocaine and amphetamines (Table 1).

**Table 1:** Patient laboratory values with reference ranges Normal lab values were obtained from UpToDate.

	Patient values	Reference ranges
Sodium level	140	136–145
Potassium level	3.5	3.5–5.0
Chloride level	109	98–106
Bicarbonate level	24.0	23–28
BUN	14	8–20
Creatinine	0.7	0.7–1.30
Glucose, blood	117	70–99 (fasting)
BUN/creatinine ratio	20	10–20
Osmolality calculated	292	275–295
Anion gap	7	7–13
Calcium level	8.6	8.6–10.2
GFR, non-African American	>60	<60
GFR, African American	>60	<60
Total protein	6.4	5.5–9
Albumin	3.5	3.5–5.5
Total bilirubin	0.4	0.3–1
SGOT	15	10–40
Alkaline phosphatase	92	30–120
SGPT	14	10–40
Magnesium level	1.9	1.6–2.6
Thyroid stimulating hormone	2.228	0.5–4
ISTAT troponin I	<0.02	≤0.04
White blood cell count	12.9	4–11
Red blood cell count	5.46	4.2–5.9
Hemoglobin	15.5	14–18
Hematocrit	48.0%	42.0–50.0%
Mean corpuscular volume	88	80–98
Mean corpuscular hemoglobin	28.3	28–32
Mean corpuscular hemoglobin concentration	32.3%	33–36
Platelet count	366,000	150,000–450,000
Red cell distribution width	13.4%	9.0–14.5
Mean platelet volume	8.3	7–9

**Figure 1:**
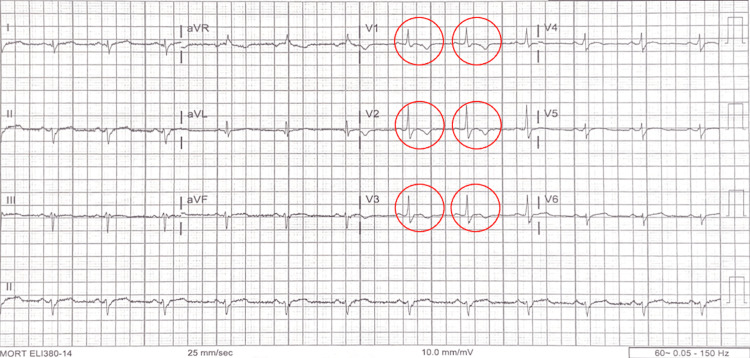
Electrocardiogram shows T-wave inversions in precordial leads V1-V3 with an associated prolonged S-wave or Epsilon wave, which is pathognomonic for ARVC.

The patient's chest X-ray was interpreted by a board-certified radiologist as having no acute pathology. The electrocardiogram (Figure 1) shows a subtle but essential morphology that could be diagnostic given the patient's presentation and continued episodes of syncope.

After a thorough history and medical record evaluation, the patient indicated that he had presented with these symptoms multiple times in the past. As a result of these episodes, the patient established care with local cardiologists. Under their management, the patient underwent an echocardiogram and transesophageal echocardiogram less than one year before this presentation to the emergency department. The echocardiogram findings: the left ventricular size and systolic/diastolic function are normal. The left ventricular ejection fraction (LVEF) is estimated at 55%. The right ventricle is not well visualized but appears mildly dilated with normal function. The tricuspid plane systolic excursion is 2 cm. There is no significant valvular disease. Unable to calculate pulmonary artery systolic pressure due to a lack of tricuspid regurgitation. Additionally, the transesophageal echocardiogram findings showed normal left ventricular size and systolic and diastolic function. LVEF is estimated at 55-60%. The right ventricle is mildly dilated in size and function. There is mild tricuspid insufficiency.

The electrocardiogram (Figure 1) shows normal sinus rhythm with no evidence of ST-segment elevation. QT intervals are within normal limits. There are notable T-wave inversions in precordial leads V1-V3 and the hallmark finding of a prolonged S-wave, or epsilon wave, which are pathognomonic for arrhythmogenic right ventricular cardiomyopathy (formerly arrhythmogenic right ventricular dysplasia) [4,5]. Of note, the patient did not have any significant changes from his prior electrocardiographic studies.

Given the patient's history of unexplained syncope, diagnostic findings on the electrocardiogram, and concerning findings on multiple modalities of the echocardiogram, the patient was admitted to the hospital for further evaluation and management. During the patient’s hospital admission, he underwent evaluation and testing by many services, including neurology, hospital medicine, electrophysiology, and cardiology. Magnetic resonance imaging (MRI) of the patient’s head was ordered to ensure no structural or pathological abnormality was causing his symptoms. After ruling out a neurologic cause, the patient began extensive cardiac imaging studies.

**Figure 2:**
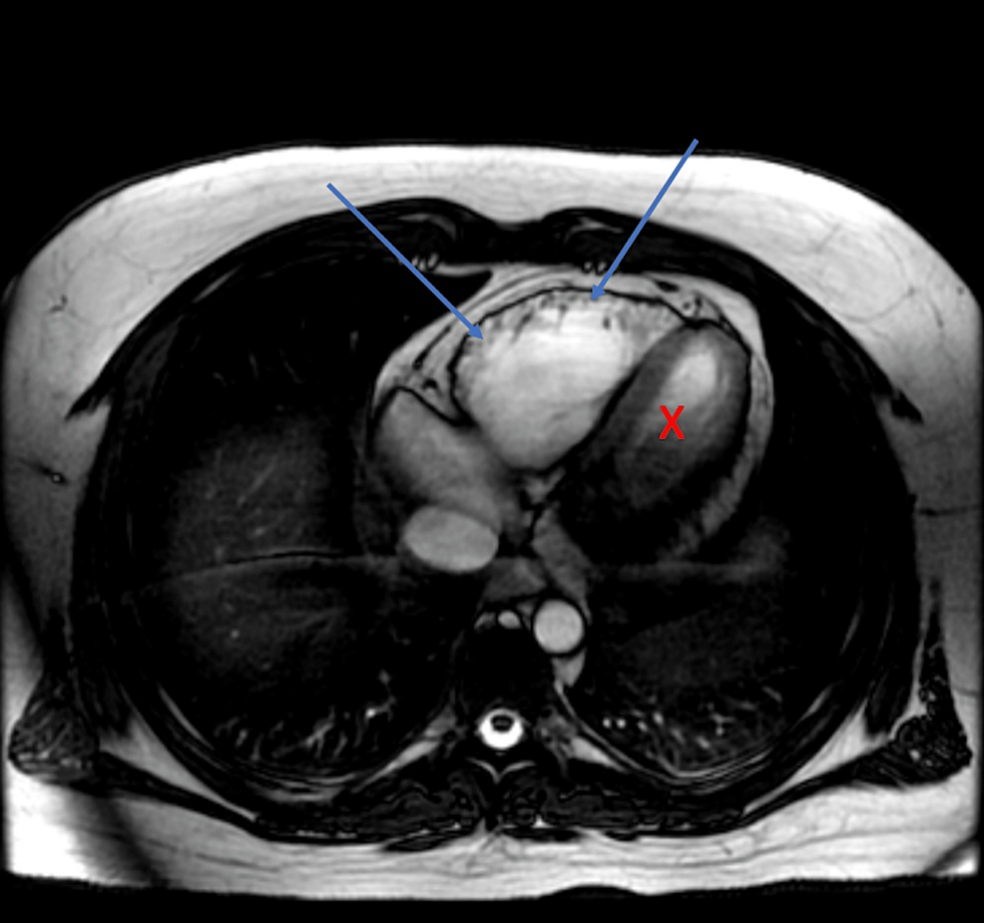
Cardiac MRI with blue arrows indicating right ventricular dilation consistent with ARVC. “X” denotes the left ventricle.

The patient was scheduled for and received both a cardiac computed tomography (CT) study and a cardiac MRI (Figure 2). The pertinent findings of the cardiac CT were normal coronary arteries and a mildly dilated right ventricle. Shortly after the cardiac CT, the patient underwent a cardiac MRI that revealed the following pertinent findings: mildly dilated right ventricle with regional wall motion abnormality and diffuse free wall delayed contrast enhancement. Mildly reduced systolic function with a right ventricular ejection fraction of 42% (RV ejection fraction >40% to ≤45% is a minor diagnostic criterion per Task Force criteria [4]). Correlate clinically for possible ARVC. Normal left ventricle size and systolic function. The left ventricular ejection fraction approximated 62%. Normal left ventricular wall thickness with no abnormalities of delayed contrast enhancement (Figure 2).

The patient was monitored continuously and started on a trial of a beta-blocker overnight. However, the beta-blocker was discontinued due to profound bradycardia during the night. Ultimately, the patient was evaluated by electrophysiology multiple times and was provided with the recommendation of a procedure to install an automated implantable cardioverter-defibrillator. However, despite numerous discussions with the electrophysiologist, the patient refused this procedure and the implantation of an internal loop recorder. He was discharged from the hospital in a hemodynamically stable and neurologically intact condition without further pre-syncopal/syncopal episodes.

## Discussion

ARVC is a sudden cardiac death disorder of the myocardium. It is not secondary to hypertensive, ischemic, or valvular heart disease [6]. ARVC is a disorder that largely affects the right ventricle. It is associated with an increased risk of sudden cardiac death and is a heritable disease [7]. ARVC is one of the most common causes of sudden cardiac death in young individuals and athletes [7]. Macroscopically, the condition shows scattered areas of fibro-fatty or fibrous involvement of the right ventricular myocardium [4].

**Figure 3:**
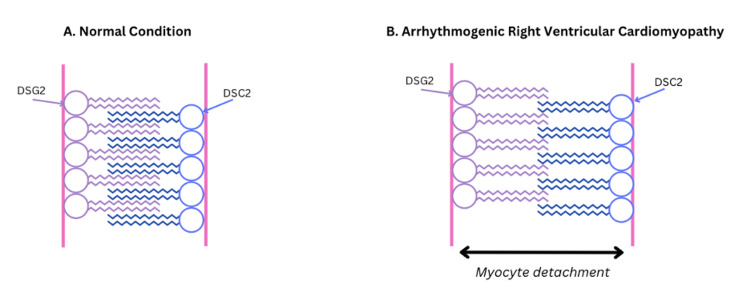
Image created and adapted from New England Journal of Medicine’s Arrhythmogenic Right Ventricular Cardiomyopathy illustration. The illustration depicts an example of how dysfunctional desmosomal proteins that contribute to the pathology of cardiomyocytes in patients with ARVC.

ARVC follows an autosomal dominant inheritance pattern with variable penetrance, and there are characteristic mutations in the genes that encode for the desmosomal proteins [8]. These desmosomal proteins are crucial for cell-to-cell adhesion [7]. The proteins that are included are desmoplakin (DSP), plakophilin 2 (PKP2), desmoglein 2 (DSG2), and desmocollin 2 (DSC2). In addition to the described mutations, ultrastructural abnormalities have been identified via myocardial biopsy in the remodeling of intercalating disks and the loss of desmosomes [7]. The replacement and incorporation of fibrofatty tissue within the right ventricle are hypothesized to cause the ventricular arrhythmias associated with this condition. Ventricular arrhythmias can arise due to a lack of integrity of the desmosomes located within the fibrofatty myocardium. Desmosomes are critical for intracellular and intercellular signal transduction [7]. As the myocardium is replaced by fibrofatty tissue, this can predispose the patient to potentially fatal ventricular arrhythmias due to the dysfunctional tissue slowing the intraventricular conduction and facilitating a macro-reentry mechanism to take place (Figure 3) [5,7].

**Figure 4:**
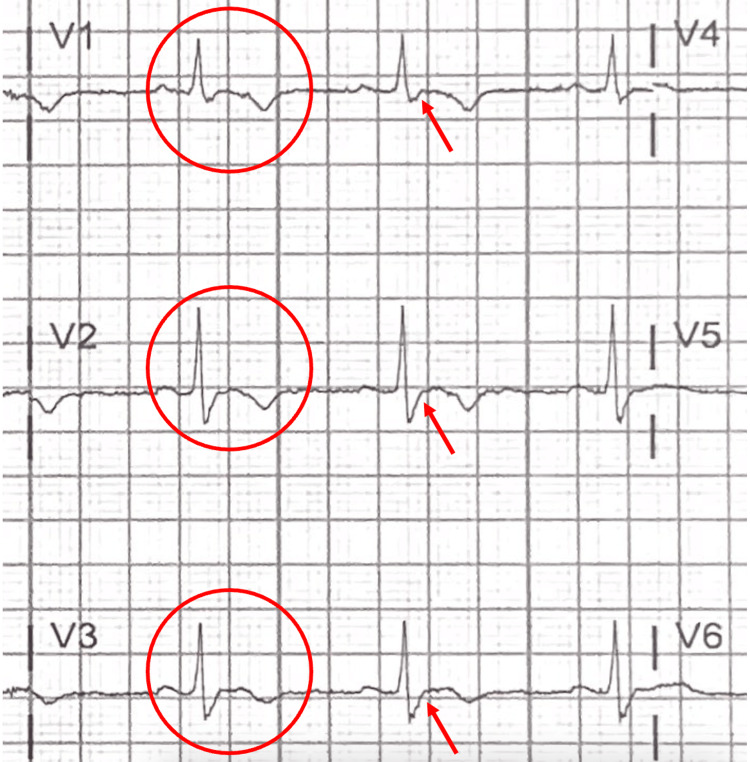
Focused image of case electrocardiogram highlighting the right precordial leads that demonstrate T-wave inversions with a prolonged duration of the terminal QRS complex or Epsilon wave.

ARVC is known to become clinically apparent in the second to fourth decade of life. Nearly 20% of deaths in young people and athletes were attributable to undiagnosed ARVC in a study from the Veneto Region of Italy [7]. The clinical presentation in this case study is typical of that of someone with ARVC. This patient presented to a local emergency department more than once with palpitations and non-exertional, near-syncope complaints. Most patient presentations with ARVC are accompanied by T-wave inversions in the right-sided precordial leads V1-V4, with an epsilon wave (indicated by the arrow in Figure 4). An epsilon wave is described as a prolonged duration of the termination of the QRS complex [5]. Damage to and development of the fibrofatty tissue within the myocardium are responsible for the conduction abnormalities seen in patients with ARVC. These abnormalities can predispose patients to ventricular arrhythmias.

If there is clinical suspicion for ARVC, the diagnosis can be made with a combination of multiple non-invasive modalities, including EKG, and imaging modalities such as echocardiogram and cardiac MRI. Cardiac magnetic resonance (CMR) is the preferred modality for patients with clinical features and other test results indicative of ARVC [1]. CMR allows experts to assess the myocardium for abnormalities, particularly those within the right ventricle. CMR is preferred over right ventriculography. CMR provides physicians with the ability to evaluate global and regional ventricular dilation, global and regional ventricular dysfunction, intramyocardial fat, late gadolinium enhancement, and focal wall thinning [4]. To standardize the diagnosis of ARVC, an international task force created diagnostic criteria for this condition and updated them in 2010 [4].

As for the clinical management of ARVC, several recommendations may be tailored to the specific patient. It is recommended that patients have lifelong cardiology follow-ups, including 12-lead electrocardiograms, echocardiograms, Holter monitoring, and exercise stress testing every one to two years [9]. Lifestyle modifications include a complete avoidance of competitive/high-endurance sports for those with a definitive diagnosis of ARVC, although there may be an exception made for low-intensity sports in some cases. Similarly, these high-intensity sports and activities should be considered to be avoided by family members with a negative phenotype or unknown genotype [9]. Given that patients with ARVC are prone to arrhythmic events, antiarrhythmic drug therapy is a mainstay of treatment. Current evidence suggests that amiodarone can either be used alone or in conjunction with beta-blocker therapy to prevent the occurrence of symptomatic ventricular arrhythmias [9]. There is also research being done on rodent models showing that preload-reducing agents, such as furosemide or nitrates, can help slow the progression of right ventricular enlargement associated with ARVC. This is not yet being implemented in clinical practice [9]. Similarly, heart failure medications such as angiotensin-converting enzyme inhibitors, angiotensin II receptor blockers, beta-blockers, and diuretics are recommended in patients with ARVC who have developed subsequent ventricular dysfunction and progressive heart failure related to the disease [9]. Finally, more invasive therapies, such as catheter ablation and implantable defibrillator placement, are also available for the treatment of ARVC. Catheter ablation is recommended in cases of ARVC with refractory ventricular tachycardia despite maximal antiarrhythmic drug therapy; however, it is not considered a long-term treatment or an alternative to implantable cardioverter-defibrillator (ICD) placement [9]. ICD placement is recommended in patients considered ‘high risk’, who have had an aborted sudden cardiac death due to ventricular fibrillation, sustained ventricular tachycardia, or severe dysfunction of the right or left ventricle. Those with ≥1 major risk factor, including syncope, nonsustained ventricular tachycardia, or moderate dysfunction of the left or right ventricle, are termed ‘intermediate risk’ and should also be considered for ICD placement [9].

## Conclusions

ARVC, as previously discussed above, is a rare cardiac condition that follows an autosomal dominant pattern of inheritance. These patients are at an increased risk of sudden cardiac death; therefore, recognizing this condition early is ideal for optimizing patient outcomes. In the case of this patient, ARVC was recognized through a constellation of symptomatology (recurrent non-exertional syncope), pathognomonic electrocardiogram findings, and echocardiogram results. Treatment recommendations for ARVC include pharmacologic therapy (beta-blockers, antiarrhythmic agents, and heart failure medications), catheter ablation, and/or implanted defibrillator therapy. The treatment for this condition is not one-size-fits-all and may be tailored based on the patient’s genetics, the severity of symptoms, and current health status. This case report serves as a good example of this principle. It highlights the subtle nature and easily overlooked presentation of the condition while displaying the dynamic nature of treatment protocols ranging from invasive procedures to commonly used medications.
